# Experiments on deformation behaviour of functionally graded NiTi structures

**DOI:** 10.1016/j.dib.2017.06.017

**Published:** 2017-06-10

**Authors:** Bashir S. Shariat, Qinglin Meng, Abdus S. Mahmud, Zhigang Wu, Reza Bakhtiari, Junsong Zhang, Fakhrodin Motazedian, Hong Yang, Gerard Rio, Tae-hyun Nam, Yinong Liu

**Affiliations:** a Laboratory for Functional Materials, School of Mechanical and Chemical Engineering, The University of Western Australia, Crawley, WA 6009, Australia; b School of Mechanical Engineering, University Sains Malaysia, 11800 Gelugor, Penang, Malaysia; c Laboratoire d’Ingénierie des Matériaux de Bretagne, Université de Bretagne Sud, Université Européenne de Bretagne, BP 92116, 56321 Lorient cedex, France; d School of Materials Science and Engineering & RIGET, Gyeongsang National University, 900 Gazwadong, Jinju, Gyeongnam 660-701, Republic of Korea

**Keywords:** Shape memory alloy (SMA), NiTi, Martensitic transformation, Functionally graded material (FGM), Pseudoelasticity, Heat treatment

## Abstract

Functionally graded NiTi structures benefit from the combination of the smart properties of NiTi and those of functionally graded structures. This article provides experimental data for thermomechanical deformation behaviour of microstructurally graded, compositionally graded and geometrically graded NiTi alloy components, related to the research article entitled “Functionally graded shape memory alloys: design, fabrication and experimental evaluation” (Shariat et al., 2017) [1]. Stress–strain variation of microstructurally graded NiTi wires is presented at different heat treatment conditions and testing temperatures. The complex 4-way shape memory behaviour of a compositionally graded NiTi strip during one complete thermal cycle is demonstrated. The effects of geometrical design on pseudoelastic behaviour of geometrically graded NiTi plates over tensile loading cycles are presented on the stress–strain diagrams.

## Specifications Table

**Table:** 

Subject area	Materials science and engineering
More specific subject area	Mechanics of materials
Type of data	Graph, figure, video
How data was acquired	Instron universal testing machine, heating and cooling devices, sputtering device, digital camera
Data format	Analysed
Experimental factors	Heat treatment temperature and testing temperature were carefully controlled.
Experimental features	Tensile tests were conducted at isothermal condition.
Data source location	The University of Western Australia, Perth, Australia.
Data accessibility	The data is with this article.

## **Value of the data**

• The data provides insights into design of shape memory components for optimum performance and a more complete picture of the experimental evidences available.
• The data can be used to design 1D microstructurally graded NiTi components with desired stress and temperature controlling windows.
• The data provides a procedure to create 2D compositionally graded NiTi structures which can exhibit unique fishtail-like deformation behaviour with enlarged temperature controlling window.
• The data provides some basic designs for creating geometrically graded shape memory structures that can be used for various engineering applications which require enlarged stress controlling window.

## 1 Data

Microstructurally graded NiTi wires have been created by means of graded temperature annealing and aging. The stress–strain diagrams of such shape memory components were obtained using tensile testing machine at various testing temperatures. Compositionally graded NiTi strips were created using sputtering deposition of a thin Ni layer and then diffusion annealing to encourage ingression of Ni through the thickness of the NiTi structure. After tensile deformation, the NiTi strips were subjected to heating and cooling cycles and the shape changes of the strip samples were recorded using a digital camera. Geometrically graded plates were created using electrical discharge machining. The deformation behaviours of a linearly tapered sample and parabolicly tapered samples under tensile loading cycles were acquired.

## 2 Microstructurally graded NiTi

**Fig. 1:**
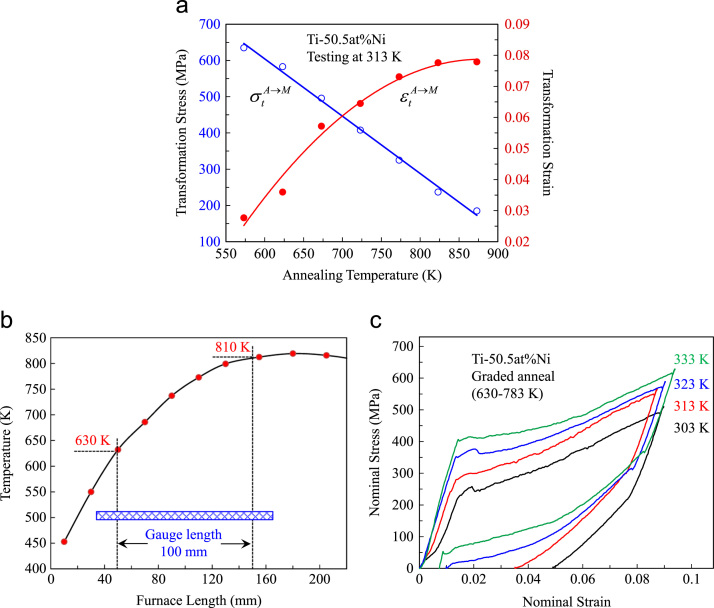
Microstructurally graded Ti-50.5 at%Ni wire created by graded annealing; (a): the effect of annealing temperature on transformation properties, (b): temperature distribution profile of the tube furnace, (c): deformation behaviour at different testing temperatures.

Microstructurally graded NiTi refers to the NiTi alloy that contains variation in microstructural characteristics, such as dislocation density, grain size and degree of crystallization within the body of the alloy [1]. This variation can be achieved by variation in heat treatment condition, such as annealing or aging temperature, and can be applied to NiTi wires along their length [2], [3] and NiTi plates across their thickness [4], [5]. It is known that the transformation properties, i.e. transformation temperature, stress and strain, are affected by heat treatment condition [6]. Fig. 1(a) shows the variation of austenite (A) to martensite (M) transformation stress and strain of Ti-50.5 at%Ni alloy with respect to annealing temperature.

### 2.1 Graded annealing

Annealing after cold working negates the effect of cold work, through recovery and recrystallization [7]. Here, Ti-50.5 at%Ni wires of 1.2 mm in diameter were used. The wires were initially solution treated at 1123 K for 1 h to eradicate any effect of prior treatments including cold working and ageing. The wires were then rolled at room temperature to 50% elongation in length. The microstructural gradient along the length of the wires was created using a tube furnace with temperature distribution profile shown in Fig. 1(b). The wires of 100 mm in length were heat treated for 1 h within a temperature field of 630–810 K along the whole gauge length as illustrated in Fig. 1(b). Fig. 1(c) shows the tensile deformation behaviour of the wire segments of 70 mm in length taken from the low temperature end of the heat treated wires, i.e. the annealing temperature range was 630–783 K. The tensile tests were carried out using an Instron testing machine at several testing temperatures. The deformation was conducted in water for elevated temperatures using a liquid bath. The strain rate was ~3×10^−4^/s. This strain rate was low enough to ignore the thermal effect associated with the latent heat of the martensitic phase transformation. A fresh sample was used for each test to avoid the effect of deformation cycles. As see in Fig. 1(c), the deformation at 303 K showed a clear presence of R-phase transformation at the early stage of deformation. The level of stress–strain curve over stress-induced transformation increased and the recovery of transformation strain improved as the testing temperature increased. Samples deformed at 323 K and 333 K exhibited good pseudoelasticity. At 333 K, the stress windows of 210 MPa and 320 MPa over forward and reverse transformations, respectively, were achieved.

### 2.2 Graded aging

**Fig. 2:**
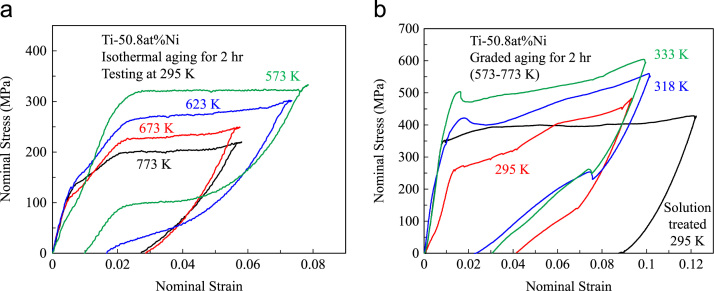
Deformation behaviour of Ti-50.8 at%Ni wires under tension; (a): isothermally aged samples at different aging temperature, (b): graded aged samples at different testing temperatures.

Ageing is applied mostly to nickel-rich alloys of >50.5 at%Ni for improving the pseudoelasticity. Ageing is affected by several parameters including alloy composition, ageing temperature and ageing time, as well as the solution treatment conditions of the alloys. Here, Ti-50.8 at%Ni wires of 0.8 mm in diameter were used. The wires were initially solution treated at 1123 K for 1 h in argon to eliminate the effects of all prior treatments. Fig. 2(a) shows the deformation behaviour of those wires after isothermal ageing at different temperatures ranging from 573 K to 773 K for 2 h. The aging temperature is shown on each curve. The samples were deformed in tension at 295 K. It is seen that by the increase of aging temperature, the stress plateau level decreased and the samples exhibited less pseudoelasticity.

Graded ageing can be done by either temperature gradient or time gradient. Here, the tensile experimental data for Ti-50.8 at%Ni wires aged over temperature gradient is presented. The wires were aged within a temperature gradient of 573–773 K along their length. The aging time was fixed at 2 h. Fig. 2(b) shows the tensile deformation behaviour of such microstructurally graded wires at different testing temperatures. The testing temperature is shown close to each curve. Also shown in this figure is the deformation behaviour of the solution treated sample, tested at 295 K. As observed, the microstructurally graded samples exhibited stress gradient over transformation stages unlike the solution treated sample.

## 3 Compositionally graded NiTi

Compositionally graded NiTi refers to the NiTi alloy that has variation in composition, i.e. Ni and Ti contents, within the body of the alloy. It is known that martensitic transformation in NiTi is sensitive to alloy composition [8]. The compositional gradient can be achieved through the thickness direction of 2D NiTi structures, such as NiTi thin plates and films [9].

In this experiment, a Ti-50.07 at%Ni sheet of 0.22 mm in thickness was used. Strips of 40 mm×5 mm were prepared from the NiTi sheet. The samples were annealed at 1123 K for 1 h in vacuum. Then, a Ni thin film of ~500 nm in thickness was deposited on the samples by means of DC magnetron sputtering under argon protection. The coated samples were then annealed at 1223 K in vacuum for 3 h to encourage diffusion of Ni into the NiTi plate. This provided a composition range of 50.07–50.8 at%Ni across the thickness direction of the strip specimens. Various levels of tensile deformation can be applied to the compositionally graded samples before the application of thermal cycles. Here, we present the experimental data for deformation behaviour of a sample during one complete thermal cycle after tensile deformation to 15%. The shape change of the strip during one cooling and heating cycle (a fishtail-like motion) was recorded by a digital camera and is presented as a video component, which accompanies the electronic version of this manuscript. To access this video component, simply click on the image visible below (online version only).

**Video S1:**
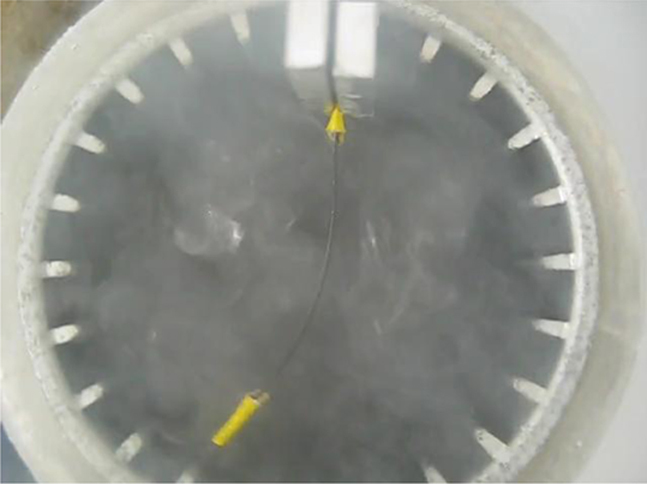
Fishtail-like deformation behaviour of a pre-strained compositionally graded NiTi strip over one thermal cycle. A video clip is available online.

**Fig. 3:**
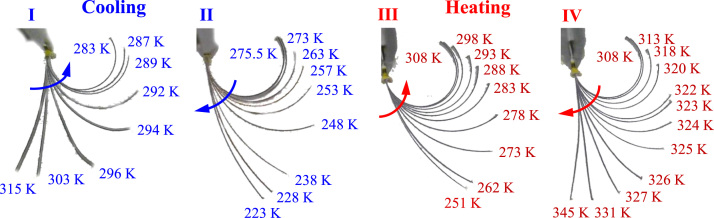
Collection of images showing macroscopic deformation behaviour of a pre-strained compositionally graded NiTi strip over one thermal cycle.

Fig. 3 shows the collection of images of that sample at different temperatures during the 20th thermal cycle. As observed, upon cooling from 315 K to 283 K, the strip progressively bended in the counter clockwise direction to a maximum curvature. Then, as the temperature was further decreased to 223 K, the strip bended back in the clockwise direction. This behaviour repeated upon heating. The strip first bended in the counter clockwise direction upon increase of temperature up to 298 K and reached to the maximum curvature. Then, it gradually curved back as heated to 345 K. This experiment presents a unique 4-way shape change during one thermal cycle.

## 4 Geometrically graded NiTi

Geometrically graded NiTi refers to the NiTi component, in which the structure geometry is purposely graded. This provides nonuniform transformation initiation in the structure, resulting in the global stress–strain variation to deviate from the original flat stress plateau. The geometrical gradient can be created along the loading direction [10], [11] or perpendicular to the loading direction [12].

**Fig. 4:**
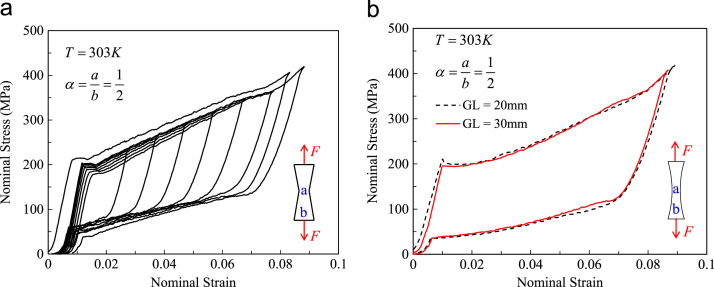
Deformation behaviour of geometrically graded Ti-50.8 at%Ni strips under tension; (a): linearly tapered, (b): parabolicly tapered.

In this section, we present the experimental data on macroscopic deformation behaviour of 2D geometrically graded NiTi structures under tension. The geometrical gradient is along the loading direction. A thin Ti-50.8 at%Ni plate of 0.1 mm in thickness was used for fabricating the geometrically graded strips with linear or parabolic edge condition by electrical discharge machining. Fig. 4 shows the deformation behaviour of such structures under tensile testing at 303 K. Here, the nominal stress is defined as the uniaxial force F divided by the maximum cross-sectional area, which corresponds to the maximum width b. The nominal strain is defined as the total elongation divided by the initial length. The width ratio α is defined as the minimum width a divided by the maximum width b. Fig. 4(a) shows the global stress–strain behaviour of a linearly tapered sample with the minimum width at the middle and the gauge length equal to 30 mm and α=1/2 under tensile loading cycles. Stress window of ~200 MPa was observed over forward transformation stage with perfect pseudoelastic behaviour over ~8% of strain. Fig. 4(b) shows the experimental data for samples with concave parabolic edges and α=1/2 with two different gauge lengths. The black dashed curve and the red solid curve represent the global stress–strain variation of the samples with the gauge length 20 mm and 30 mm, respectively.
